# The Modification of Polyurethane Foams Using New Boroorganic Polyols (II) Polyurethane Foams from Boron-Modified Hydroxypropyl Urea Derivatives

**DOI:** 10.1155/2014/363260

**Published:** 2014-01-22

**Authors:** Iwona Zarzyka

**Affiliations:** Department of Organic Chemistry, The Faculty of Chemistry of Rzeszow University of Technology, Powstańców Warszawy 6, 35-929 Rzeszow, Poland

## Abstract

The work focuses on research related to determination of application possibility of new, ecofriendly boroorganic polyols in rigid polyurethane foams production. Polyols were obtained from hydroxypropyl urea derivatives esterified with boric acid and propylene carbonate. The influence of esterification type on properties of polyols and next on polyurethane foams properties was determined. Nitrogen and boron impacts on the foams' properties were discussed, for instance, on their physical, mechanical, and electric properties. Boron presence causes improvement of dimensional stability and thermal stability of polyurethane foams. They can be applied even at temperature 150°C. Unfortunately, introducing boron in polyurethanes foams affects deterioration of their water absorption, which increases as compared to the foams that do not contain boron. However, presence of both boron and nitrogen determines the decrease of the foams combustibility. Main impact on the decrease combustibility of the obtained foams has nitrogen presence, but in case of proper boron and nitrogen ratio their synergic activity on the combustibility decrease can be easily seen.

## 1. Introduction

Rigid polyurethane foams-based research derives from the need of change of their properties, such as physical (density, water uptake, dimensional stability, and combustibility), mechanical (compressive strength and brittleness), and electric ones (thermal conductivity). One of the main aspects of polyurethane materials manufacturing improvement is the elimination of toxic compounds from the production process and their replacement with environmental friendly ones [1, 2]. Furthermore, works on foams combustibility decrease are currently undergoing—this particular property considerably reduces their employment. The foams' compositional change, aimed at the mentioned decrease, usually causes the deterioration of their functional attributes. Therefore, it seeks to obtain foams with reduced combustibility and fixed mechanical properties [3–6]. It can be reached by the foams cross-link density increase or through azacyclic compounds or nitrogen content in foams. Flame retardants containing phosphorus, boron, and nitrogen are more and more commonly used as well [7, 8].

Bearing in mind the polyurethane foams properties modification issue, the work focuses on research oriented at the determination of new boroorganic polyol application possibility in rigid polyurethane foams production. The use of boron in foam compositions results in more organized polymer structure, which significantly and positively influences on their mechanical strength. Furthermore the presence of boron in foams structure decreases their combustibility [9, 10].

In this paper, new polyols were obtained by means of ecofriendly substrates, for example, in N,N′-bis(2-hydroxypropyl)urea (BHPU) esterified by boric acid (BA) reaction with propylene carbonate (PC) excess. Rigid, foamed polyurethane materials were obtained and tested by means of the above-mentioned polyols and 4,4′-diphenylmethane diisocyanate. The influence of production way of the polyol on properties of new foams was investigated. Then attributes of foams obtained from boron-modified and unmodified hydroxypropyl urea derivatives were juxtaposed and some foams' properties were compared with other reference foams.

## 2. Materials and Methods

### 2.1. Syntheses

#### 2.1.1. Reactions of BHPU with BA—Method 1

In a round bottom flask 176 g (1 mole) of BHPU was obtained according to the recipe [11] and 124 g (2 moles) of BA was placed. Then it was heated in an oil bath, with the flask open and continuous stirring to 110°C and set stable for approximately 8 hours. The progress of reaction was monitored by determination of acid number and reaction mixture weight loss.

#### 2.1.2. Reactions of BHPU with BA—Method 2

176 g (1 mole) of BHPU, 124 g (2 moles) of BA, and 400 cm^3^ of xylene were inserted into a round-bottomed flask fitted with a mechanical stirrer, Dean-Stark trap, and reflux-condenser. Then it was heated to 100°C and set stable until 2 moles of water were azeotropically distilled. The xylene was distilled off in a rotary evaporator and the reaction's product (EBU) was dried off to a stable weight in a vacuum dryer at 90°C and 0.09 MPa.

#### 2.1.3. Reactions of BHPU Esterified by BA with Excess of PC

In a three-necked round bottom flask 132 g (0.5 mole) of BHPU (EBU) esterified by boric acid and the appropriate amount of PC (pure, Fluka, Switzerland) was placed to reach the molar ratio of reagents of 1 1 : 6, 1 : 12 and 1 : 18, and 6.21 or 8.28 g of potassium carbonate (12.42–16.56 g K_2_CO_3_/mole EBU; 0.09–0.12 mole K_2_CO_3_/mole EBU) was added. It was equipped with a reflux-condenser accompanied by a drying tube and a mechanical stirrer and was heated to 180°C (Table 1) during continuous stirring. The reaction's progress was monitored by determination of unreacted PC percentage in reaction mixture [12].

#### 2.1.4. Reactions of BHPU with Excess of PC

In a round-bottomed flask 88 g (0.5 mole) of BHPU and such an amount of PC that initial mole ratio of reagents will be, respectively, 1 : 6, 1 : 12 and 1 : 18, and 8.28 g of potassium carbonate that is 16.56 g/mole BHPU; 0.12 mole/mole BHPU were placed. It was equipped with a reflux-condenser accompanied by a drying tube and a mechanical stirrer and was heated to 160°C during continuous stirring. The reaction' progress was monitored by determination of unreacted PC percentage in reaction mixture.

#### 2.1.5. Obtaining Foamed Polyurethane Materials

10 g of proper hydroxypropyl urea derivative, 0.1 g of a surfactant (L-6900 Silicon, Momentive, USA), 1.5–4.6 wt% of triethylamine (TEA, catalyst, pure, Avocado, Germany), and 2–6 wt% of water (Table 4) were inserted into a 250 cm^3^ polyethylene cup. Having thoroughly mixed all the components, a properly calculated amount of 4,4′-diphenylmethane diisocyanate (with a triisocyanate additive [MDI], pure, Merck, Germany) was added and later on experimentally corrected. When an isocyanate was added up, it was vigorously stirred until creaming occurred. Times of creaming, expanding, and drying of the foams were measured. With the aim of the next tests, after 48 hours of foams seasoning in room temperature, fittings were cut out of them.

### 2.2. Methods of Analysis

Hydroxyl number (HN) of the obtained polyols was determined with the use of acetic anhydride and pyridine [13].

Thermogravimetric analysis (DTG and TG) of the above-mentioned products were made in ceramic pans in the following registration conditions: temperature range 20–600°C, registration time 60 min, sample weight 1-2 mg, and atmosphere-nitrogen, with the use of a derivatograph Termowaga TGA/DSC, Mettler.

The polyols' molar masses (number-average (${\overset{-}{M}}_{n}$), weight average (${\overset{-}{M}}_{w}$), *z*-average (${\overset{-}{M}}_{z}$)) were determined with the use of a gel chromatograph Viscotec T60A equipped with RI detector (of refractive index). Separation was performed by means of two independent columns: PSS SDV (of 7.8 mm × 300 mm dimensions), accompanied by TSK deposits of 100 and 1000 Å pore diameter and the following recording conditions: 25 ± 0,1°C, eluent flow rate: 1 cm^3^/min, injected sample size: 20 *μ*dm^3^, polymer concentration in solution: 5 mg/cm^3^, analysis time: 30 min, and eluent: THF (distilled from above sodium); calibration was performed on the basis of typical polystyrene and branched references.

The elemental analysis (C, H, and N) of foams was made by means of an element analyzer Vario EL III C, H, N, S, and O, manufactured by Elementar. Boron content tests in foams were performed by means of spectrophotometric method [14].

The following polyurethane foams' properties were determined: apparent density [15], water uptake [16], dimensional stability [17], glass transition temperature (by means of DSC method), thermal stability measured by weight loss during monthly annealing at 150 and 175°C (one month is required to acquire stable weight), compressive strength [18], thermal conductivity [19], and oxygen index [20].

The microscopic observation of foams was performed at optical microscope Nikon Eclipse LV100 POL, camera Digital Sight DS-5Mc, objective 2,5 or 5 × L Plan. Microscopy measurements were performed in the Biophysical Laboratory of the Department of Physic of Rzeszow University of Technology. The Laboratory has been constructed in the frame of UE Polish Integrated Regional Operation Program.

Measurements of DSC foams were made with the use of DSC822e calorimeter, manufactured by Mettler Toledo, at 20–200°C temperature range and heating rate 10 deg/min in nitrogen atmosphere [21].

Tests on thermal conductivity and heat capacity coefficients were made at room temperature and by means of Isomet 2114 (Applied Precision, Slovakia), portable measuring apparatus. A rebate probe was used in the experiments as well; it required sample of 40 mm diameter and 80 mm length [19].

Oxygen index experiment, based on minimal oxygen concentration in oxygen and nitrogen mixture, in which a sample of tested material set in measuring column lights a fire longer than 3 minutes, was performed with the use of a device destined for the oxygen index determination (Fire Testing Technology Limited, UK) [20].

## 3. Discussion on the Results

### 3.1. Boroorganic Polyols Production and Properties

**Scheme 1:**
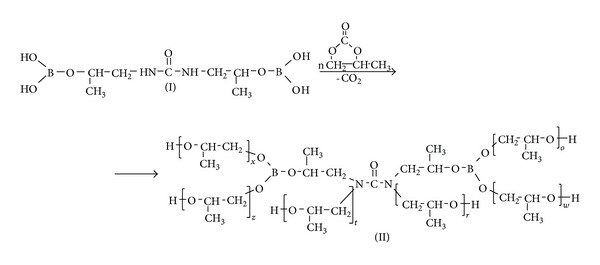


New boroorganic polyols (II)—boron-modified hydroxypropyl urea—were obtained 8 as a result of EBU (I) with 6-, 12- and 18-molar PC excess reactions at 180°C and in the presence of a catalyst—potassium carbonate (Table 1) (also see Scheme 1, where *n* = 6, 12, and 18).

It should be noted that EBU was produced by means of two methods, which has been described in detail in the work [22]. In the first one BHPU was subject to direct boric acid reaction without any solvent, heating the reagents up to 130°C. In the latter case BHPU was esterified by boric acid in xylene environment, azeotropically distilling off a proper amount of water. The obtained EBU was used in reaction with excess of PC, thus obtaining two polyol series: A polyols with EBU (according to method 1.—Table 1, synth. 1–3) and B polyols with EBU (according to method 2.—Table 1, synth. 4–6). At the same time, C polyols synthesis was made, but in this case they had no boric groups (Table 1, synth. 7–9).

### 3.2. Properties of the Obtained Polyols

Thermal stability of the derivatives was measured by means of thermogravimetric method. Polyols obtained without boric acid presence (P1C–P3C) are characterized by slightly higher thermal stability than boron-modified polyols; their 5-% weight loss occurs at 130–230°C temperature range (Table 2), whereas 5-% weight loss of modified polyols occurs at 125–150°C temperature range [22]. A and B polyols display similar thermal stability.

Average molar masses of hydroxypropyl urea derivatives were determined by means of gel chromatography (Table 3). Polyols mass increases along with the increase of PC excess used in reactions. Molar masses weight average of A polyols fall into 487–603 g/mole range, while molar masses of B polyols are a bit lower—441–586 g/mole [22]. C polyols, unmodified by boric acid, have the lowest masses (Table 3, numbers 1–3). Moreover, hydroxyl numbers (HN) of the obtained polyols were also determined (Table 1).

### 3.3. Characteristics of the Foaming Process

Foaming of hydroxypropyl urea derivatives modified and unmodified by boric acid were performed with the presence of MDI (as an isocyanate component). The initial attempts showed that 2–6% of water is the best amount, as far as foaming is depending on polyol type and on amount of oxypropylene units contained in it. The smallest amount of water (2 wt%) was applied in manufacturing foams with boron-unmodified polyols (Table 4, P1C–P3C).

Similar relationships can be observed in case of catalyst presence—the smallest amount of it is used, when the oxypropylene units in a polyol is as low as possible (Table 4).

Creaming time of foamed compositions falls into 10–37 sec range—in addition, compositions obtained with polyols with no boron present (C polyols) are characterized by the shortest creaming time, while the longest one sticks to A polyols. Such relations can occur in case of expanding and drying times (Table 4), but foams from C polyols (boron-unmodified) are almost completely dry after the end of expanding time (Table 4, P1C–P3C).

### 3.4. Polyurethane Foams Properties

**Figure 1:**
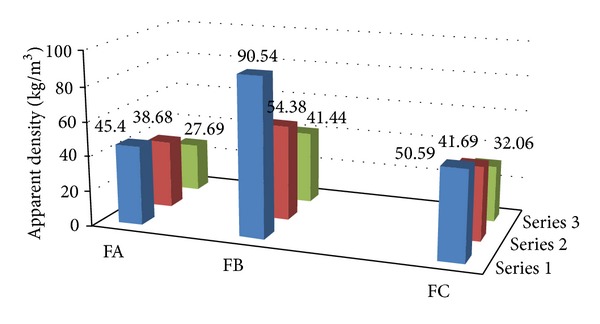
Apparent density of polyurethane foams.

Apparent density of the boron-modified foams obtained from A polyols falls into a range typical for polyurethane foams 28–44 kg/m^3^ (Figure 1, FA). It has been observed that the density decreases along with the increase of oxypropylene units in a polyol, so with the decrease of boron content in a foam.

Similar relationships can be seen in case of foams obtained from B polyols, but their density values are significantly higher (41–90 kg/m^3^). The boron-unmodified foams are characterized by density falling within 32–51 kg/m^3^—analogous to foams obtained from A polyols. It should be noticed, however, that foams densities obtained from C polyols increase together with the increase of the amount of oxypropylene units in a polyol component, whereas in cases of A and B polyols a reverse effect can be recognized (Figure 1).

**Figure 2:**
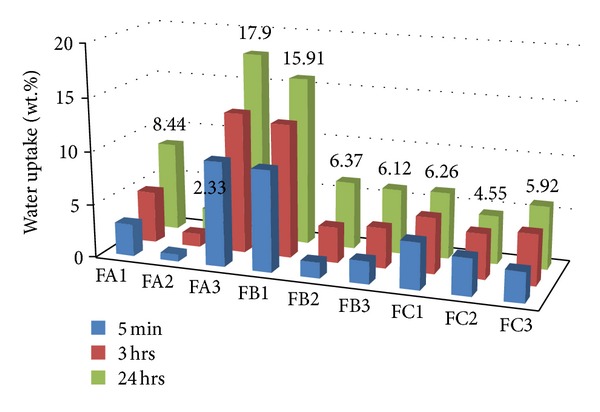
Water uptake of polyurethane foams.

Water uptake after 24-hour in-water exposition at room temperature [16] of all boron-unmodified foams is alike and comprises into 4.6–6.3 wt% range. It is also usually smaller than in the case of the boron-modified ones (6.1–17.9 wt%). One exception are FB2 foams (Figure 2) obtained from A polyol of average boron content, which reveal the smallest (the best) water uptake (2.3 wt%). This uptake of the modified foams depends on the boron content in given foams (the amount of oxypropylene units) and undergoes changes, but they are not regular ones.

**Figure 3:**
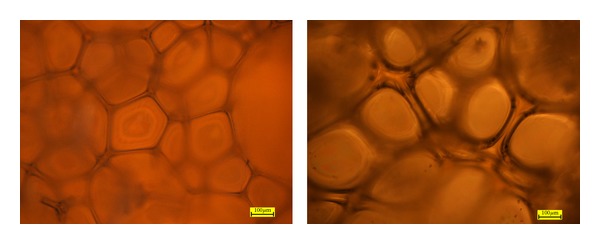
Morphology of foams: (a) FA1 and (b) FB1, magnification 10x.

Observations of the foams under an optical microscope have evidenced that their structure contains mainly closed pores of a regular distribution. In the case of foams obtained from A polyols, wall thickness of the pores is 15–20 *μ*m and size of the pores is put in the range of 0.12–0.33 mm. The foams obtained from B polyols have pores of slightly thicker walls of 30–50 *μ*m but similar sizes of 0.11–0.30 mm. Furthermore, it was noted that pores of the foams prepared with the contribution of A polyols have sharp-edged shapes, while those of the foams obtained from B polyols are more rounded (Figure 3).

Insertion of boron into structure of foams increases their dimensional stability (Table 5), while the boron content changes' impact on regular dimensional foam modification has not been observed so far. Yet, unmodified foams' dimensional changes fall into 4–20% linear values (Table 5, FC1–FC3), whereas the same changes, in case of all boron-modified foams, do not exceed 3% linear values. What is more, slightly smaller modifications apply to foams obtained from B polyols (Table 5; FA1–FA3, FB1–FB3).

Glass transition temperature (*T*_*g*_) of the boron-modified, foamed polyurethane materials, determined by the DSC method, ranges at 127–162°C (Table 6). The glass transition temperature values of foams obtained from proper A and B polyols are comparable (Table 6; FA1 and FB1, FA2 and FB2). The glass transition temperature values of foams obtained from C polyols (the boron-unmodified) is lower than in the corresponding modified foams (Table 6).

It has also been noticed that glass transition temperature of the modified foams decreases along with the increase of oxypropylene units in a polyol (e.g., the decrease of boron content), while the glass transition temperature of unmodified foams increases together with the increase of the oxypropylene units in a polyol (Table 6).

The glass transition temperature of all foams is higher than room temperature, which allows qualifying the obtained foams as the rigid ones [23].

Thermogravimetric analysis showed high thermal stability of the obtained polyurethane foams (Table 6). 5% weight loss of foams obtained from A polyols occurs only at temperature range 210–230°C (Table 6). As for DTG curve, one can notice that in case of foams obtained from the polyol component with the highest boron content (Table 6, FA1), 3 extremes occur—the first at 210°C, the second at 240°C, and the third one at 315°C. The first extremum appears due to boron groups' decomposition [24], the second due to carbamate groups' decomposition, and the third one due to urea groups' decomposition [25]. Foams obtained in the presence of PA2 polyols show two DTG curve extremes—the first at 260°C (simultaneous decomposition of boron and carbamate groups) and the second at 315°C (Table 6, FA2).

Foams obtained from B polyols are characterized by slightly smaller thermal stability; 5% weight loss occurs in temperature around 190°C (Table 6; FB1 and FB2), whereas the foams DTG curve shows two extremes—as in the preceding example—at 260°C and 310°C (Table 6, FB1 and FB2).

The thermal stability of all boron-modified foams is higher in comparison with the unmodified ones but is considerably higher in case of A polyol foams than in case of B polyol ones (Table 6). 5% weight loss of the unmodified foams occurs in 180°C. Yet, on the boron-unmodified foams DTG curve only one extremum can be observed—at 260°C (Table 6; FC1 and FC2).

In order to test the thermal resistance of the obtained polyurethane foams, they were exposed to 150 and 175°C temperature until stable weights occurred (approximately 30 days) (Table 7, Figure 3). As for 150°C temperature, weight losses lower than 10% apply to almost every foam type obtained from B polyols and those foams get from A polyols, which are characterized by higher boron content (Table 7; FB1–FB3 and FA1, FA2). This means that the above-mentioned foams may be applied at 150°C temperature.

**Figure 4:**
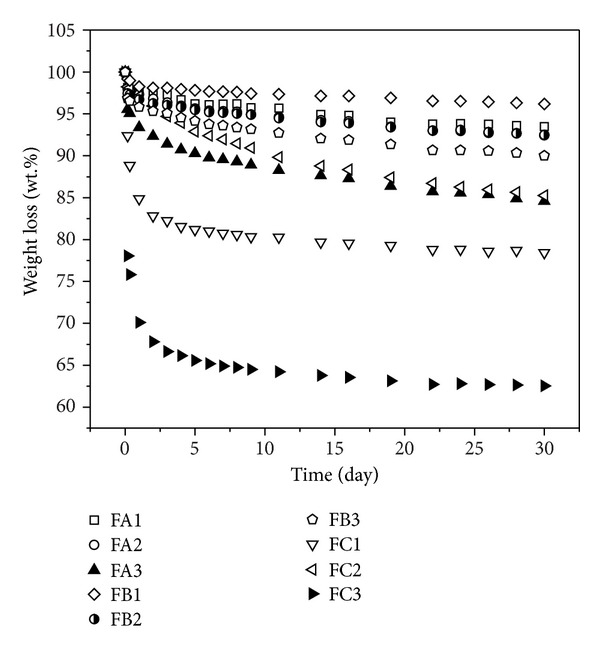
Comparison of heat resistance of the boron-modified and unmodified foams on the basis of their 30-day annealing at 150°C.

On the other hand, weight losses of the unmodified foams reach up to 20% (Table 7; FC1–FC3; Figure 4).

Due to large weight loss of the obtained foams in the presence of PA3 polyols, reaching 15.4%, and foams from PB3 polyols amounting to 10%, such foams are not taken into consideration in case of other properties' investigation. Foams obtained from C polyols (FC1 and FC2), however, were tested despite large weight losses because they are foams of reference.

In temperature reaching up to 175°C such losses are considerably higher, while the smallest weight losses (16–26%) apply to foams from B polyols (Table 7; FB1–FB3). The other modified (from A polyols) and unmodified foams (from C polyols) display deficiencies amounting even up to 40% (Table 7).

In general, the unmodified foams weight losses are much larger than the modified ones but we can notice that in higher temperatures differences between weight losses of modified and unmodified foams being smaller.

The obtained foams were strength tested, performing these tests before annealing and after 150°C annealing (but only those that showed weight loss below 10%). All foams were compressed parallel to the direction of their growth, measuring their compressive strength accompanied by 10% deformation.

Foams obtained from A polyols possess compressive strength ranging from 0.1 to 0.22 MPa (Table 8; FA1–FA3). The strength change does not depend directly neither on boron content in given foams nor on foam's density (Figure 1, Table 10; FA1–FA3). Foams of average boron content and average apparent density (38.7 kg/m^3^; Figure 1; FA2) display the highest compressive strength—0.22 MPa (Table 8, FA2).

Foams compressive strength increases after temperature exposition at 150°C—the largest growth occurs in case of foams with the highest boron content. All foams A, annealing at 150°C, show almost identical compressive strength, which does not depends on boron content (Table 8; FA1 and FA2).

Foams obtained from B polyols possess higher compressive strength: 0.28–0.54 MPa (Table 8; FB1–FB3). The influence of the boron content on the foams compressive strength can be clearly seen here; along with the boron content decrease, the foams' compressive strength deteriorates as well. It should also be noted that the foams obtained from B polyols possess higher apparent density, what impacts on their compressive strength (Figure 1 and Table 8; FA1–FA3 and FB1–FB3). Juxtaposing the strength of A and B polyol foams of almost identical density (Figure 1; FA2 and FB3); it is visible that the B ones display higher strength accompanied by lower boron content (Table 8; FA2 and FB3). After successful temperature exposition at 150°C, the B foams compressive strength increases, but only in case of foams with the lower boron content (Table 8; FB1–FB3).

Comparing the compressive strength of the unmodified, 0.02–0.04 MPa (Table 8, FC1–FC3), and modified foams, 0.10–0.54 MPa (Table 8; FA1–FA3 and FB1–FB3), the boron content has significant impact on the increase of this property. Taking into account the influence of the apparent density on the foams' strength (Figure 1 and Table 8; FC2, FA2 and FB3; FB2 and FC3; FC1 and FA3), it can be stated that the above-mentioned increase is 5–15-times greater.

What is more, the compressive strength of the obtained foams was compared with the same property of typical foams (0.24 MPa) obtained with the use of Rokopol RF-55 and MDI of similar apparent density (36.9 kg/m^3^) [26]. It has been claimed that the boron-modified foams obtained from B polyols (Table 8, FB3) possess higher compressive strength (0.28 MPa). Nevertheless, their compressive strength is lower than that of condensation polyurethanes with different hard segments [27].

Insulation parameters have been tested and due to these experiments it has been claimed that the thermal conductivity decreases along with the increase of foam boron content (Table 9; FA1 and FA2; FB1 and FB2). Foams obtained from A and B polyols containing lower amount of boron have the identical thermal conductivity value (Table 9; FA2 and FB2). On the other hand, foams of higher boron content, obtained from B polyols, display lower thermal conductivity, 0.0308 (W/(m·K)), than foams obtained from A polyols, 0.0321 (W/(m·K)) (Table 9, FA1); thus, they possess better heat-insulating properties. Furthermore, it has been observed that the thermal conductivity of these foams is lower than 0.035 (W/(m·K)), so it is typical for polyurethane foams to be employed as insulation materials [28].

Having examined the insulation parameters of the modified and unmodified foams, it has been said that the thermal conductivity of the modified ones is lower (Table 9).

What is more, in case of the boron-modified foams obtained from A and B polyols the thermal conductivity increases together with the volume heat capacity (Table 9; FA1 and FA2; FB1 and FB2); thus, the better insulation, the worse heat accumulation [28].

The volume heat capacity of the unmodified foams is usually lower than the corresponding modified ones (Table 9), but when the thermal conductivity increases, the volume heat capacity decreases, so it is the opposite situation than in case of the modified foams.

**Figure 5:**
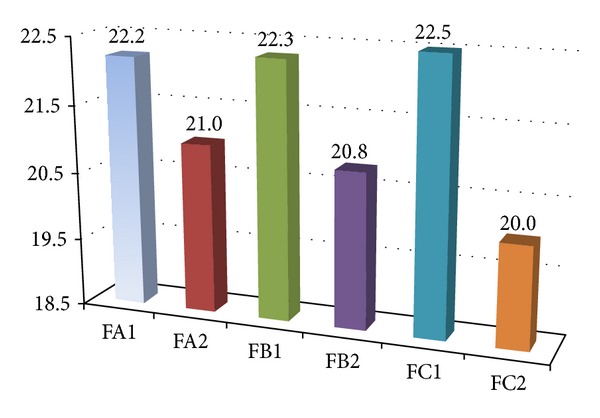
Foam oxygen index values (OI).

In order to illustrate the influence of the boron content on the decrease of the combustibility of the obtained polyurethane foams, their OI has been determined (Figure 5). Comparing the OI values of the obtained foams (Figure 5; FA1 and FA2; FB1 and FB2) with the OI value of a typical foams obtained in the presence of Rokopol RF-55 and MDI amounting to 19.6% [10], a clear impact of the boron presence on the combustibility decrease can be seen. OI value increases together with boron content in foams (Figure 5, FA1 and FA2; FB1 and FB2). On the basis of the OI value it can be said that foams obtained from A polyols (Figure 5, FA1 and FB1), as well as foams of higher boron content obtained from B polyols (Figure 5, FB1), are self-extinguishing (OI higher than 21%) [29]. It has also been proved that foams obtained from B polyols of higher boron content (Figure 5, FB1), having been fired, extinguish spontaneously, whereas foams of lower boron content (Figure 5, FB2) die down in approximately 1 minute. Such phenomenon was not observed in case of foams obtained from A polyols.

Comparing the OI values of the boron-modified and unmodified foams influence of the boron presence on the OI value of foams obtained in the presence of a polyol component, obtained from larger excess of PC, so containing lower amount of boron (Figure 5; FA2, FB2 and FC2), has been observed.

The determined OI values show that nitrogen in foams (urea groups) should also be taken into consideration because it causes the decrease of the combustibility. When the nitrogen content is higher, foams obtained from polyols obtained with 6-molar PC excess of boron-modified and unmodified have almost identical OI values (Figure 5; FA1, FB1 and FC1), but a bit higher possess the unmodified ones. It has been pointed out that in case of 9 wt% nitrogen content in foams (Table 10), the boron presence has no impact on the decrease of their combustibility. The boron-modified foams obtained from polyols with more oxypropylene units (Figure 5; FA2, and FB2), so lower nitrogen content (about 8 wt%), display higher OI value in comparison with the unmodified ones (Figure 5; FA2, FB2, and FC2). Thus, synergic boron and nitrogen activity related to the foams combustibility decrease can be observed. Nevertheless, the OI value of such foams is lower than those with higher nitrogen content (Figure 5).

## 4. Recapitulation and Conclusions

1. Rigid, foamed polyurethane materials displaying good dimensional and thermal stability are obtained in the presence of hydroxypropyl urea derivatives of boron-modified.
2. The insertion of boron into the foams' structure causes that they can be applied even at 150°C.
3. The polyurethane foams boron modification evokes a significant compressive strength increase, as well as influences their insulation values in comparison with both foams obtained from the unmodified urea derivatives and other foams obtaining from typical polyol components.
4. Nitrogen has a main impact on the combustibility decrease of the obtained foams, but in case of proper boron and nitrogen ratio, their synergic activity on the combustibility decrease can be easily seen.
5. Foams of better properties were obtained with the presence of a polyol component obtained as a result of the N,N′-bis(2-hydroxypropyl)urea esterified by boric acid reaction accompanied by xylene.

## Figures and Tables

**Table 1:** Polyol synthesis conditions and composition.

Synth.	Initial molar ratio* BHPU : BA : PC	Amount of K_2_CO_3_ (mole/BHPU mole)	Temp. (°C)	Reaction time (h)	Formal molar ratio BHPU : *x* in product	HN (mg KOH/g)	Polyol sign
1.	1 : 2 : 6	0.12	180	8	1 : 5.6	622 ± 21	PA1
2.	1 : 2 : 12	0.12	180	14	1 : 11.1	540 ± 18	PA2
3.	1 : 2 : 18	0.12	180	22	1 : 16.2	469 ± 19	PA3
4.	1 : 2 : 6	0.12	180	13.25	1 : 5.9	687 ± 22	PB1
5.	1 : 2 : 12	0.12	180	17	1 : 10.4	626 ± 20	PB2
6.	1 : 2 : 18	0.12	180	20	1 : 15.7	523 ± 17	PB3
7.	1 : 0 : 6	0.12	160	17	1 : 5.7	526 ± 18	PC1
8.	1 : 0 : 12	0.12	160	22	1 : 11.3	467 ± 15	PC2
9.	1 : 0 : 18	0.12	160	32	1 : 16.2	388 ± 12	PC3

*x*: oxypropylene unit.

*In case of syntheses 1–6 EBU was used directly.

**Table 2:** Thermal stability of hydroxypropyl derivatives of urea.

Number	Polyol type	*T*_5%_ (°C)	*T*_10%_ (°C)	*T*_20%_ (°C)	*T*_50%_ (°C)	*T*_max⁡_ (°C)
1.	PC1	130	155	185	215	220
2.	PC2	230	265	280	320	300
3.	PC3	170	185	215	260	270

*T*_*x*%_: temperature, in which *x*% weight loss occurs.

**Table 3:** Molar masses of some obtained polyols determined by means of GPC method.

Number	Polyol type	${\overset{-}{M}}_{n}$ (g/mole)	${\overset{-}{M}}_{w}$ (g/mole)	${\overset{-}{M}}_{z}$ (g/mole)	HN (mg KOH/g)
1.	PC1	382	388	392	526 ± 18
2.	PC2	412	418	423	467 ± 15
3.	PC3	531	538	542	388 ± 12

**Table 4:** Impact of polyol composition on foaming process.

Polyol type	Foam type	Composition (g/100 g of polyol)			Foaming process		
		Isocyanate*	Water	Catalyst**	Time (s)		
					Creaming	Expanding	Drying
PA1	FA1	268	3	1.73	32	90	10
PA2	FA2	184	4	2.30	37	91	90
PA3	FA3	144	2	2.30	27	77	120
PB1	FB1	300	4	2.59	15	90	10
PB2	FB2	200	6	4.61	16	45	45
PB3	FB3	160	3	4.03	20	70	68
PC1	FC1	220	2	1.54	10	10	1
PC2	FC2	144	2	2.11	15	34	1
PC3	FC3	112	2	2.16	12	83	1

*4,4′-diphenylmethane diisocyanate; **triethylamine; creaming time: time from start of stirring to start of growth; expanding time: time from start of growth to obtaining final dimensions; drying time: time from the end of foam growth to lack of adhesion of powder materials.

**Table 5:** Dimensional stability of polyurethane foams.

Foam type	Dimensional stability (linear %) in 150°C					
	Length		Width		Thickness	
	After 20 h	After 40 h	After 20 h	After 40 h	After 20 h	After 40 h
FA1	1.96 ± 0.03	1.96 ± 0.05	2.86 ± 0.09	2.86 ± 0.09	2.68 ± 0.23	2.65 ± 0.23
FA2	0.84 ± 0.03	0.84 ± 0.04	1.11 ± 0.03	1.11 ± 0.03	0.99 ± 0.07	0.99 ± 0.09
FA3	1.32 ± 0.11	1.76 ± 0.13	0.13 ± 0.03	1.39 ± 0.50	0.45 ± 0.03	0.90 ± 0.04
FB1	0.64 ± 0.05	0.64 ± 0.06	0.00 ± 0.00	0.68 ± 0.02	0.00 ± 0.00	0.00 ± 0.00
FB2	0.15 ± 0.02	0.75 ± 0.05	0.52 ± 0.02	2.09 ± 0.06	0.00 ± 0.00	1.06 ± 0.11
FB3	1.91 ± 0.12	2.23 ± 0.67	0.54 ± 0.04	0.45 ± 0.02	0.48 ± 0.06	0.68 ± 0.09
FC1	18.40 ± 1.50	16.89 ± 1.28	16.11 ± 1.93	15.98 ± 1.79	3.91 ± 0.42	8.59 ± 0.76
FC2	7.87 ± 0.26	9.55 ± 0.23	5.51 ± 0.43	8.27 ± 0.55	6.25 ± 0.29	10.42 ± 0.72
FC3	8.28 ± 0.82	9.08 ± 1.01	14.58 ± 1.38	15.23 ± 1.54	15.94 ± 1.60	19.81 ± 2.02

**Table 6:** Polyurethane foams thermal stability.

Foam type	*T*_5%_ (°C)	*T*_10%_ (°C)	*T*_20%_ (°C)	*T*_50%_ (°C)	*T*_max⁡_ (°C)	*T*_*g*_ (°C)
FA1	210	235	255	310	210, 240 and 315	155
FA2	230	250	250	305	260 and 315	136
FB1	185	210	235	300	260 and 310	162
FB2	190	235	245	300	265 and 315	127
FC1	180	220	240	420	260	86
FC2	180	200	240	440	260	119

*T*_*x*%_: temperature, in which *x*% weight loss takes place.

**Table 7:** Foams weight loss following the 30-day annealing.

Foam type	Foam weight loss (wt%) following the annealing in a given temperature (°C)	
	150	175
FA1	6.52 ± 0.01	23.61 ± 0.91
FA2	7.54 ± 0.23	23.86 ± 0.01
FA3	15.42 ± 0.67	41.03 ± 0.58
FB1	3.68 ± 0.48	16.00 ± 0.11
FB2	7.32 ± 0.51	21.95 ± 0.91
FB3	9.65 ± 0.75	25.85 ± 1.30
FC1	21.57 ± 0.13	29.47 ± 0.06
FC2	24.74 ± 0.02	26.75 ± 0.04
FC3	37.48 ± 0.35	41.06 ± 1.02

**Table 8:** Compressive strength of the obtained polyurethane foams.

Foam type	Compressive strength *σ*_*M*_ (MPa)	Compressive strength after annealing at 150°C	
		*σ*_*M*_ (MPa)	Growth (%)
FA1	0.10 ± 0.004	0.34 ± 0.014	340
FA2	0.22 ± 0.007	0.36 ± 0.012	164
FA3	0.18 ± 0.007	—	—
FB1	0.54 ± 0.022	0.92 ± 0.016	70
FB2	0.31 ± 0.019	0.41 ± 0.013	132
FB3	0.28 ± 0.012	0.32 ± 0.007	114
FC1	0.04 ± 0.002	—	—
FC2	0.04 ± 0.002	—	—
FC3	0.02 ± 0.001	—	—

**Table 9:** Insulation parameters.

Foam type	Thermal conductivity, λ (W/(m·K))	Volume heat capacity, *C*_*ρ*_ · 10^−6^ (J/(m^3^·K))
FA1	0.0321 ± 0.0007	0.0798 ± 0.0008
FA2	0.0375 ± 0.0016	0.0885 ± 0.0030
FB1	0.0308 ± 0.0003	0.0685 ± 0.0008
FB2	0.0375 ± 0.0003	0.0784 ± 0.0008
FC1	0.0564 ± 0.0001	0.0583 ± 0.0003
FC2	0.0382 ± 0.0003	0.0868 ± 0.0001

**Table 10:** Content of boron and nitrogen in the obtained foams.

Foam type	Boron content (wt%)	Nitrogen content (wt%)
FA1	0.833	9.15
FA2	0.616	8.12
FB1	0.851	8.95
FB2	0.703	8.07
FC1	0	8.98
FC2	0	7.66
